# Rare Case of Milk Cyst in the Breast, under the Care of M. Jobert, Hôpital Saint Louis

**Published:** 1845-01

**Authors:** 


*Rare case of Milk Cyst in the Breast, under the care of* M. Jobert, *Hôpital Saint Louis.*—A young woman, aged 26, of good constitution, was admitted into hospital on account of a tumour of the right breast: it had attained the size of the head of a child nine months old, was globular, without change of colour in the super-incumbent skin, was without pain, resistant, hardly compressible, but evidently fluctuating, yielding the sensation of a vessel very full of fluid; it was moveable at the base, and could even be somewhat displaced under the integuments. The skin covering it was thin and flabby, rolling under the fingers, and yielding a sensation similar to that which would be produced by granular bodies situated below the cellular tissue; two large veins ran vertically over its surface. The tumour was situated exactly at the internal side of the nipple and mammary gland; it was pendulous, and in the course of its development had pushed the gland upwards and outwards, the latter, however, partially adhered to it. There was no transparency on examining it with a candle. On pressing the nipple, milk could be sqeezed out; this, however, was also the case in the other breast, as the woman had had a child two months previously. The following is the patient's own statement regarding it:—She has had four children, and the tumour first appeared six years ago, after her first delivery; it was small at first, and on pressing it, milk appeared at the nipple, even a long period after the delivery. After her second confinement the tumour increased in size, as it did also after her two subsequent; pressure on the tumour always caused milk to escape by the nipple, so that from the period of her first confinement the milk had never entirely disappeared from that breast. The tumour, however, acquired its greatest development only after the last delivery; at that period it was much larger than it is now, and was accompanied with a painful feeling of distention, which was only relieved by squeezing out the milk from the breast by means of repeated pressure: this, at the same time, caused the tumour to diminish in size. Such is the history of the disease. M. Jobert diagnosed a cyst. But no one suspected the nature of the fluid it contained. Previous to operating, M. Jobert made a small exploratory puncture by means of a trochar; to his astonishment there immediately followed a jet of pure milk, a glassful was allowed to escape, which was afterwards sent to the laboratory of the Faculty for analysis. It was inodorous, and had the slight opaline tint of healthy milk. As M. J. had no expectation of being able to cure the tumour by evacuating the fluid and injecting irritating substances into the cyst, owing to the enormous size of the pouch, which rendered it impossible that its thin and flabby parietes would contract sufficiently to adhere, and as the patient urgently requested to be relieved of it in the speediest way, he determined to remove it by operation. By means of a bistoury he made an oval incision, and dissected out the flap so as to remove part of the loose superabundant tissue; he then opened the cyst, and nearly a pint of milk escaped; the entire removal of the pouch was accomplished by dissection by means of scissors, bistoury, and forceps.

The cyst adhered, at its upper and external part, to the mammary gland, it had also formed attachments to some small neighbouring tumours; the whole of the diseased parts were completely dissected and removed. The wound having been washed, and several small vessels tied, milk was perceived to escape from a point at the bottom of the wound corresponding to the mammary gland. It proceeded from some lactiferous vessels which communicated with the cyst, and had been divided. The wound united by the first intention.

On subsequent dissection of the parts, several remarkable, as well as unexpected conditions were found. The principal cyst was of large size, thin, and polished, like serous cysts in general; in its centre, there was an ulcerated spot of the size of half-a-crown, with raised irregular edges ; its base appeared somewhat infiltrated with milk. On examining attentively this serous pouch, several small openings were discovered ; through one of these a probe was passed, which led to a group of granulations in the mammary gland which had been removed along with the cyst. This passage was discovered to be an enlarged lactiferous vessel, and there were many others. It was now evident that several enlarged and hypertrophied lactiferous vessels proceeded from various granular groups of the mammary gland, and communicated with the cyst; and that, through them, the milk which had been met with, had been poured. This also satisfactorily explained how milk should have escaped from the nipple when pressure was made on the tumour, and its diminution in size. The lateral cysts were three or four in number; one of them had attained the size of an egg, the others that of a nut, they all contained milk, and communicated with groups of granulations in the gland.

Mention has been made of milk cysts in the breast in several works, and also by Dupuytren in his lectures. But a general description of these tumours has nowhere been given, and the details of individual cases which we meet with are usually so slight, that we may safely assert that the nature of these cysts has yet to be studied. A paper on this subject, however, has appeared from the hand of the celebrated Sir Benjamin Brodie of London. The general description given by Sir B., is entirely confirmed by the case of M. Jobert. It is impossible, indeed, after mature deliberation, to doubt that these cysts owe their origin to a dilatation of some of the lactiferous vessels, and that the ulceration met with in the case of M. J., was but the commencement of a fungous process which occurs at a certain period of the disease, exhibited by the facts stated by Sir Benjamin. According to the latter, it is absolutely necessay to remove the whole breast ; but it is probable that, in the case of M. J., a cure may be effected by the removal of the cyst alone. It remains to be seen, however, whether the small tubes yielding milk at the bottom of the wound will be obliterated, or whether they will again produce a new cyst ; the result of the case alone will show.—*Lon. and Ed. Month. Jour., from Annales de Thérapeutique.*

